# Comparative study on molecular epidemiology of measles H1 outbreak and sporadic cases in Shandong Province, 2013–2019

**DOI:** 10.1186/s12864-022-08492-x

**Published:** 2022-04-14

**Authors:** Suting Wang, Changyin Wang, Xiaodong Liu, Yao Liu, Ping Xiong, Zexin Tao, Meng Chen, Qing Xu, Li Zhang, Aiqiang Xu

**Affiliations:** Shandong Center for Disease Control and Prevention, No. 16992 Jingshi Road, Jinan, 250014 People’s Republic of China

**Keywords:** Measles virus, H1, Phylogeny, Molecular epidemiology

## Abstract

**Background:**

Measles caused by measles virus (MeV) is a highly contagious viral disease which has also been associated with complications including pneumonia, myocarditis, encephalitis, and subacute sclerosing panencephalitis. The current study isolated 33 strains belonging to 2 groups, outbreak and sporadic strains, in 13 cities of Shandong province, China from 2013 to 2019. Comparison of genetic characterization among 15 outbreak strains and 18 sporadic strains was performed by analyzing nucleotide sequences of the C-terminal region of N protein gene (N-450).

**Results:**

All 33 stains belonged to genotype H1. The outbreak strains and sporadic strains distributed crossly in phylogenetic tree. Sequences alignment revealed some interesting G to A transversion which changed the amino acids on genomic sites 1317, 1422, and 1543. The nucleotide and amino acid similarities among outbreak isolates were 98–100% (0–10 nucleotide variations) and 97.7–100%, respectively; They were 97.3–100% and 96.6–100%, respectively for sporadic isolates. Evolutionary genetics analysis revealed that the mean evolution rates of outbreak and sporadic isolates were 1.26 N 10^− 3^ and 1.48 N 10^− 3^ substitutions per site per year separately, which were similar with corresponding data before 2012. Local transmission analysis suggested that there were three transmission chains in this study, two of them originated from Japan. Outbreak cases and sporadic cases emerged alternatively and were reciprocal causation on the transmission chains.

**Conclusions:**

Our study investigated the phylogeny and evolutional genetics of MeV during a 7-year surveillance, and compared epidemic and genetic characteristics of outbreak strains and sporadic strains. These results underscore the importance of evolutionary study alongside with sporadic cases in discovering and tracing possible outbreaks, especially in the stage of measles elimination.

**Supplementary Information:**

The online version contains supplementary material available at 10.1186/s12864-022-08492-x.

## Introduction

Measles caused by measles virus (MeV) is a highly contagious viral disease which has also been associated with complications including pneumonia, myocarditis, encephalitis, and subacute sclerosing panencephalitis. It is one of the leading causes of death among young children worldwide, with a fatality rate as high as 15% in children [1]. Even though a safe and cost-effective vaccine is available, there were still 140,000 measles deaths globally in 2018, mostly among children under 5 years of age [2]. MeV is an enveloped virus with a nonsegmented, negative-sense RNA genome that is 15,894 nucleotides in length. It belongs to the genus *Morbillivirus* in the *Paramyxovirinae* sub-family. MeV have 24 genotypes recognized to date (A, B1 –B3, C1 – C2, D1 – D11, E, F, G1 – G3 and H1 – H2) [3, 4] and there are four predominant measles genotypes currently circulating worldwide: D8, B3, H1 and D4 [5]. MeV is genotyped using carboxy-terminal 450 nucleotides of N-region (N-450), the most variable sequence in the MeV genome, or the entire protein-coding region of the H gene [6].

The Measles and Rubella laboratory Network (LabNet) of mainland China has been established in 2001 to monitor progress toward mortality reduction and elimination of measles. Besides serologic testing, another important function of the network is to support the genetic characterization of currently circulating measles viruses. Based on the data from LabNet, the epidemiology characterization of MeV had changed a lot in China in recent years, that is, the seasonal distribution had delayed the epidemic peak, the age distribution had changed over the years, and the incidence of measles varied dramatically among different provinces [7]. In Shandong province, from the early beginning of 2013 to the first half of 2016, the incidence of measles had been increasing with the morbidity ranging from 0.15/million to 0.45/million. Then, the incidence decreased rapidly till 2019 when the morbidity of measles was only 0.019/million. Measles patients did not emerge as an outbreak pattern in Shandong Province. Most of them were sporadic cases since the second half of 2016. Plausible causes of incidence change need to be investigated.

It has been well accepted that the molecular characterisation of virus strains is essential for monitoring MeV transmission and the progress of elimination efforts [8]. N-450 region is a useful target for investigating possible correlation among concurrent measles cases [9]. According to a previous study on N-450 region, changes in measles transmission occurred in China. The transmission pathways in 2011–2012 have been interrupted and many new transmission lineages of unknown origin occurred in 2013–2016 [10]. In this study, an effort was made to elaborate the genetic correlation between the sporadic and outbreak strains during 2013–2019.

## Results

### Epidemiological information

**Fig. 1:**
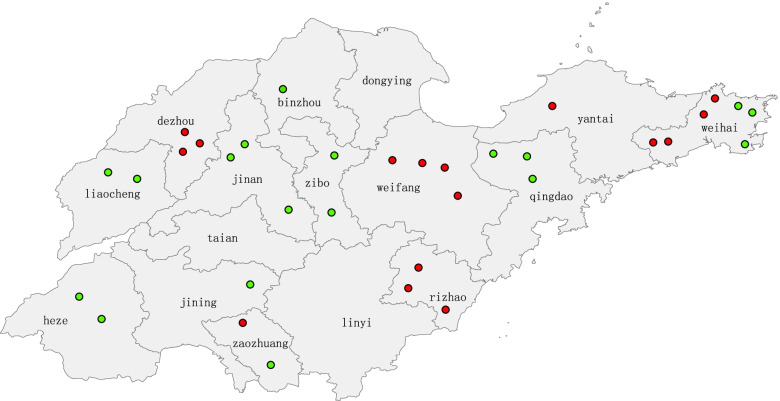
Geographic distribution of measles virus isolates. One marker represents one isolate. The red makers are isolates from outbreak cases, and the green ones are from sporadic cases

The outbreak cases were collected from 8 outbreaks involving 6 cities. Four sporadic cases were collected from 2 of the 6 cities above. All the outbreak cases lived in infected area for more than 21 days, however 2 of them lived less than 3 months. No imported outbreak cases from other provinces or countries were reported. Out of the 15 outbreak cases, 10 cases were males and 5 were females. In terms of age, 8 cases were children under 15 years old and 7 were adults. For 18 sporadic cases selected in this study, 9 were males and 9 were females; 6 were children under 15 years old and 12 were adults. Except 2 cases, which were floating population from other provinces, 31 cases involved in this study were resident population. All cases which clinical information were available had fever and rash, most of them accompanied with cough, less with catarrhus and conjunctivitis, only 4 cases accompanied with other symptoms (pneumonia or bronchitis). Vaccination information was available from 8 out of 15 outbreak cases. Of these, 4 were not vaccinated, 3 received only one dose and 1 received two doses of measles-containing vaccine. Vaccination information was also available from 8 out of 15 sporadic cases. Of these, 6 were not vaccinated, 2 received only one dose of measles-containing vaccine (Table 1). In this study, we tried to make sure there were both outbreak strains and sporadic strains in the same isolate time (Table 1). The outbreak and sporadic strains we selected from 13 cities which have certain consistency in geographical distribution. Isolate areas and quantities are shown in Fig. 1.

**Table 1:** Clinical symptoms and vaccination history of measles cases together with the isolate time

	code	fever	rash	cough	catarrhuss	Conjunctivitis	Other complications	measle vaccine	isolate time (year)
Outbreak cases	MVi/ShanDong.PRC/24.14/1	Y	Y	Y	Y	–	–	2 dose	2014.6
	MVi/ShanDong.PRC/9.15/1	Y	Y	Y	Y	Y	–	not available	2015.2
	MVi/ShanDong.PRC/9.15/2	Y	Y	Y	–	Y	–	not available	2015.2
	MVi/ShanDong.PRC/9.15/4	–	–	–	–	–	–	not available	2015.2
	MVi/ShanDong.PRC/9.15/5	–	–	–	–	–	–	not available	2015.2
	MVi/ShanDong.PRC/14.15/1	Y	Y	Y	–	–	–	0 dose	2015.3
	MVi/ShanDong.PRC/14.15/4	Y	Y	Y	Y	Y	–	not available-	2015.3
	MVi/ShanDong.PRC/14.15/6	Y	Y	Y	Y	–	–	not available-	2015.3
	MVi/ShanDong.PRC/22.15/1	Y	Y	Y	–	Y	–	0 dose	2015.3
	MVi/ShanDong.PRC/20.16/1	Y	Y	Y	–	Y	–	1 dose	2016.4
	MVi/ShanDong.PRC/20.16/2	Y	Y	–	–	–	–	0 dose	2016.3
	MVi/ShanDong.PRC/20.16/3	Y	Y	–	–	Y	–	not available	2016.3
	MVi/ShanDong.PRC/18.19/1	Y	Y	Y	Y	–	pneumonia	0 dose	2019.4
	MVi/ShanDong.PRC/18.19/2	Y	Y	Y	Y	Y	pneumonia	1 dose	2019.4
	MVi/ShanDong.PRC/18.19/3	Y	Y	Y	Y	–	pneumonia	1 dose	2019.4
Sporadic cases	MVi/ShanDong.PRC/17.13/1	Y	Y	Y	Y	Y	–	1 dose	2013.4
	MVi/ShanDong.PRC/17.13/2	–	–	–	–	–	–	not available	2013.4
	MVi/ShanDong.PRC/16.13	Y	Y	Y	Y	–	–	1 dose	2013.4
	MVi/ShanDong.PRC/24.14/2	–	–	–	–	–	–	not available	2014.5
	MVi/ShanDong.PRC/24.14/3	Y	Y	Y	–	Y	–	not available	2014.5
	MVi/ShanDong.PRC/16.14	Y	Y	Y	Y	Y	–	0 dose	2014.3
	MVi/ShanDong.PRC/9.15/3	Y	Y	–	–	–	–	0 dose	2015.2
	MVi/ShanDong.PRC/14.15/2	Y	Y	Y	Y	Y	–	0 dose	2015.2
	MVi/ShanDong.PRC/14.15/3	Y	Y	–	–	–	–	not available	2015.3
	MVi/ShanDong.PRC/14.15/7	Y	Y	Y	Y	Y	–	not available	2015.3
	MVi/ShanDong.PRC/28.15/2	Y	Y	Y	–	Y	–	0 dose	2015.5
	MVi/ShanDong.PRC/17.15	–	–	–	–	–	–	not available	2015.3
	MVi/ShanDong.PRC/22.15/2	Y	Y	Y	–	–	–	0 dose	2015.4
	MVi/ShanDong.PRC/16.16	Y	Y	Y	–	Y	–	0 dose	2016.3
	MVi/ShanDong.PRC/26.16	Y	Y	Y	Y	Y	bronchitis	not available-	2016.4
	MVi/ShanDong.PRC/17.19/1	–	–	–	–	–	–	not available-	2019.4
	MVi/ShanDong.PRC/17.19/2	Y	Y	–	–	–	–	not available-	2019.4
	MVi/ShanDong.PRC/17.19/3	Y	Y	–	–	–	–	not available	2019.4

The dashes represent there were no according symptoms here

### Sequences alignment

All genetic changes in the Shandong isolates in our study were base substitutions, with no deletion, insertions, or frame-shift mutations, but some interesting mutations were observed. Sequences AB824116 which is a reference sequence obtained from GenBank and MVi/ShanDong.PRC/17.13/1, MVi/ShanDong.PRC/16.14, and MVi/ShanDong.PRC/17.15 which were isolated from sporadic cases, together with MVi/ShanDong.PRC/24.14/1, MVi/ShanDong.PRC/9.15/1, and MVi/ShanDong.PRC/9.15/5 which were obtained from outbreaks, generated G to A transversions on sites 1317, 1422, 1543 (nucleotide positions referring to measles reference sequence) at the same time comparing to other sequences in our study. All these transversions leaded to amino acid changes. Interestingly, these sequences were isolated in different years during 2013–2015 but with 100% similarity with each other. There were also unique mutation on nt position 1417 of MVi/ShanDong.PRC/26.16 and 1581 of MVi/ShanDong.PRC/16.14, both of which induced amino acid changes. MVi/ShanDong.PRC/26.16 and MVi/ShanDong.PRC/16.14 were both the sporadic case sequence. The nucleotide and amino acid similarities among the 15 outbreak isolates were 98–100% (0–10 nucleotide variation) and 97.7–100%, respectively. For sporadic isolates, they were 97.3–100% and 96.6–100%, respectively. All 33 Shandong isolates were clustered into two groups, sporadic group and outbreak group. The p-distance between the 2 groups was 0.008.

### Genotype and phylogenetic analysis

**Fig. 2:**
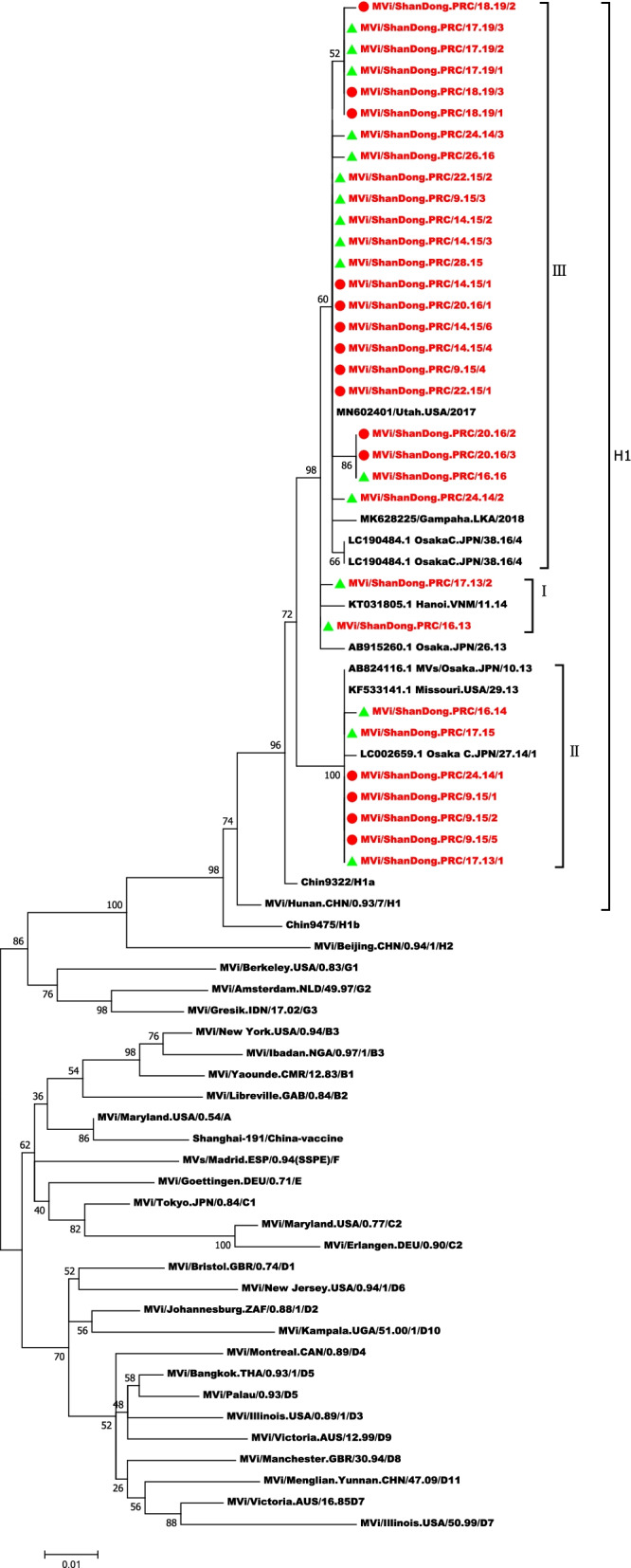
Molecular phylogenetic analysis by Maximum Likelihood method. The evolutionary history was inferred by using the Maximum Likelihood method based on the General Time Reversible model. The tree with the highest log likelihood (− 2525.36) is shown. The percentage of trees in which the associated taxa clustered together is shown next to the branches. Sequences labeled by red font are Shandong strains; Sequences labeled by red circles are outbreak strains and green triangles are sporadic strains from Shandong

N-450 PCR products of the 33 viral isolates were purified and sequenced. All 33 viral isolates together with 9 reference sequences obtained from GenBank were genotyped as H1, using WHO reference strains sequences by Maximum Likelihood tree. Further phylogenetic analysis was conducted among 33 Shandong strains and 9 references sequences (Fig. 2). All the sequences of H1 can be divided into 3 clusters (cluster I, II and III). The evolutionary tree analysis revealed that the outbreak case sequences and the sporadic case sequences did not cluster separately. Similarly, isolates from different cities cross-distributed, not clustered by city. AB824116.1, MVi/ShanDong.PRC/17.13/1, MVi/ShanDong.PRC/17.15, MVi/ShanDong.PRC/24.14/1, MVi/ShanDong.PRC/9.15/1, and MVi/ShanDong.PRC/9.15/5 which had the totally same sequence and formed a distinctive cluster. It should be noted that 9 outbreak strains, MVi/ShanDong.PRC/9.15/5, MVi/ShanDong.PRC/9.15/2, MVi/ShanDong.PRC/9.15/1, MVi/ShanDong.PRC/9.15/4, MVi/ShanDong.PRC/14.15/1, MVi/ShanDong.PRC/14.15/4, MVi/ShanDong.PRC/14.15/6, MVi/ShanDong.PRC/22.15/1, and MVi/ShanDong.PRC/20.16/1, which were all isolated in the same year of 2015, belonged to different branches.

### Evolution rate and molecular clock analysis

Eight model combinations were compared using AICM calculation, finally, a strict clock with an constant size model was employed with the lowest AICM value (2328.872). Although different evolution rates were observed for some different branches, most branches had the similar evolution rate. The mean evolution rate for all 33 Shandong isolates with 9 reference isolates was 1.833 N 10^− 3^ substitutions per site per year. The mean evolution rate for 15 outbreak and sporadic isolates was 1.26 N 10^− 3^ and 1.48 N 10^− 3^ substitutions per site per year, respectively.

**Fig. 3:**
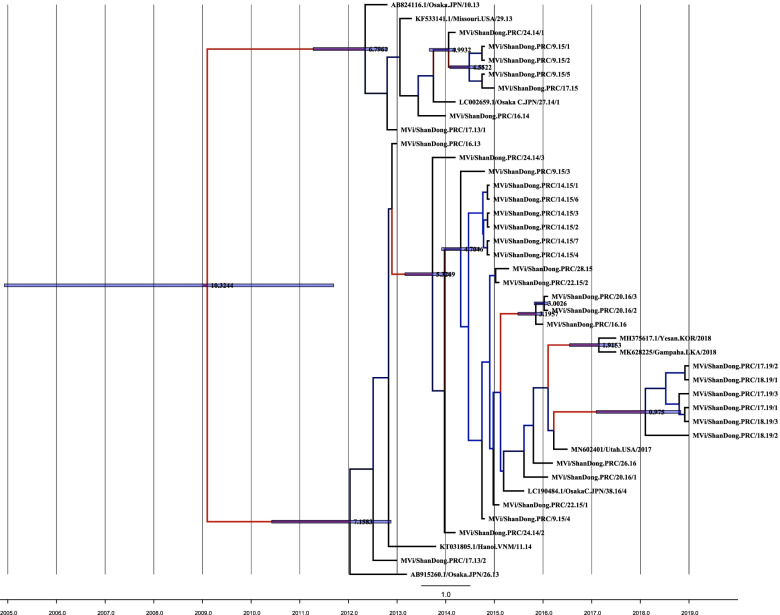
Maximum clade credibility tree representing the measles genotype H1 evolutionary history inferred by using Bayesian evolutionary analysis. The tree has branch lengths in time units and is depicted on a time scale. The uncertainty (95% highest posterior density intervals) for the node times is indicated with blue bars. Branches labels supported by posterior probabilities are indicated by using a blue-black-red (slow-average-high) color scheme. Rate variation among branches is indicated by using a blue-black-red (slow-average-high) color scheme too

The molecular clock phylogenetic tree was also conducted (Fig. 3). Shandong isolates didn’t segregated into a single cluster. Outbreaks and sporadic strains were dispersed in molecular phylogenetic trees. Figure 3 shows the maximum clade credibility tree that summarizes the evolutionary history estimated by using a strict molecular clock. The most recent common ancestor (MRCA) of all H1 lineages (clusters I and II) dated back to 2009(1896 to 2012). Cluster II had the same older MRCA with cluster I (2012) with overlapping credible intervals (2013 to 1896 versus 2013 to 1963).

The file generated for the phylogenetic analysis with BEAST software were labled in additional files (Additional file 1).

### Phylogeographic analysis

**Fig. 4:**
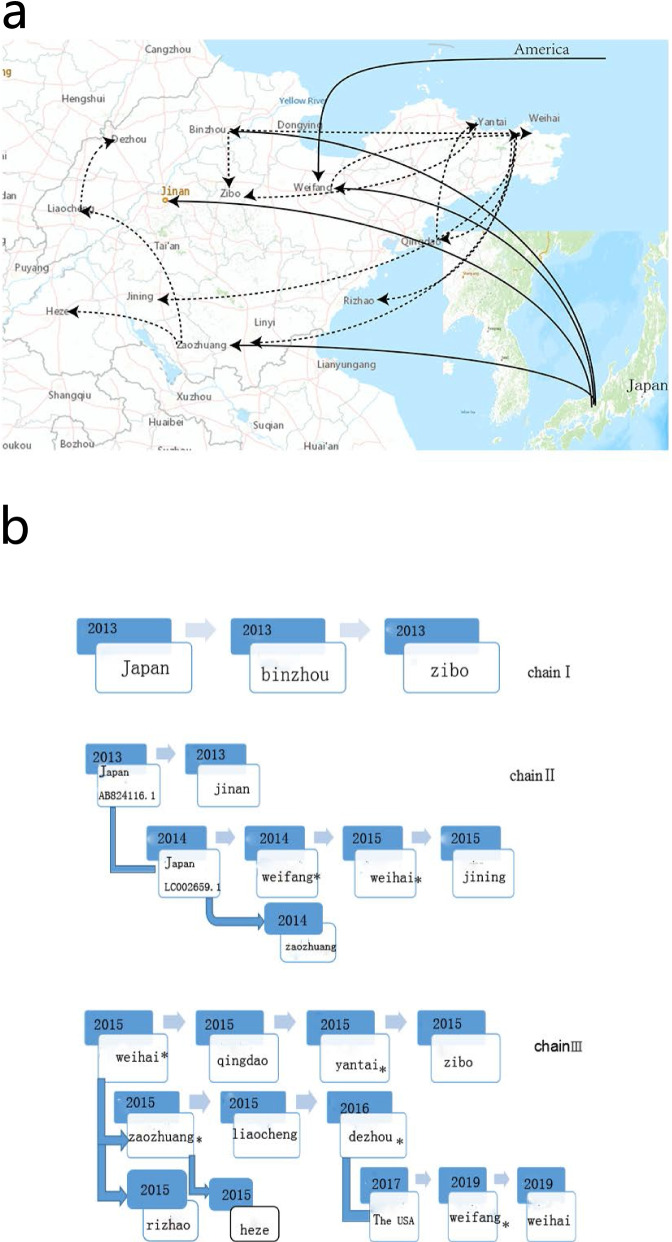
Geographic distribution and inferred dynamics of measles virus strains. The map 4a was reconstructed using the ArcGIS (http://www.esri.com/), and was identical to the original image created by the SPREAD and GoogleTM Earth. Arrows indicate the inferred routes of spread of measles virus strains. 4b is a flow chart of three propagation chains based on 4a. The number represented the time when strains arrived. The migration routes of the strains transmitted in Shandong Province were figured by dashed lines. Continuous Lines figure the routes of imported strains

The geographic dispersal of measles cases was conducted with the N-450 sequences and was shown in Fig. 4a and b (Additional files 2 and 3). We analyzed the results together with the Phylogenetic trees, finally we defined three main transmission chains (chain I, II and III) according with the clusters on Phylogenetic trees. Two of them originated from Japan, and one was local transmission of Shandong province. On the chain I, the virus strain crossed Japan and arrived in Binzhou in 2013 then propagated to Zibo in the same year. On the chain II, the first viruse strain originated from Japan arrived Jinan in 2013 then exported to the USA. One year later, another Japanese strain which has only one nucleotide difference with the first one on the chain II arrived in Weifang and Zaozhuang in2014, then propagated to Weihai and terminated in Jining in 2015. On the chain III, the viruse strain originated from Weihai in 2015, then followed two distinct routes: i) to Qingdao, passing by Yantai and terminated in zibo in the same year. ii) to Rizhao and Zaozhuang, passing by Lliaocheng and Heze in 2015, then terminated in Dezhou in 2016. Till 2017, the virus strain with the same sequence with the viruses on the chain III emerged in the USA, and arrived Weifang in 2019,then terminated in Weihai in the same year.

## Discussion

China, including Shandong Province, is now in the phase of accelerated measles control. Although from 2016, measles incidence remained at a low level until 2021, and most were sporadic cases, there were still outbreaks occurred occasionally in Shandong before 2020. Many factors are associated with occurrence of measles outbreak, such as accumulation of susceptible children, seasonal factors, density of population and so on [11–15]. In this study, efforts were made to explore the factors causing measles outbreaks from another perspective. We analyzed the phylogenetic relationship among sporadic and outbreak isolates from 13 prefectures of Shandong provinces from 2013 to 2019 to study possible correlation and gennetic divergences Local transmission conducted with the N-450 sequences were also assayed to find out the disseminate relationship between the outbreak and sporadic strains.

As reported previously, measles virus has high genetic stability and there is very little variation in the N sequences of viruses isolated from the same chain of transmission [16]. Moreover, very little genetic changes revealed from the same genotype that were collected several years apart [17, 18]. Similarly, in our study, no matter outbreak sequences or sporadic sequences isolated from different cities or different years have high identities of nucleotide and protein. And the distances between the two group are only 0.008. These results demonstrate measles strains circulating in Shandong kept high stability between the outbreak and sporadic strains. However, there were some interesting mutations found in our study. As shown in the Results section, sequences AB824116.1, MVi/ShanDong.PRC/17.13/1, MVi/ShanDong.PRC/17.15,MVi/ShanDong.PRC/24.14/1, MVi/ShanDong.PRC/9.15/1 and MVi/ShanDong.PRC/9.15/5 generated G and A transversions on three sites (site 1317, 1422, 1543) and induced amino acid changes on three corresponding sites.

On phylogenetic tree, the outbreak case sequences and the sporadic case sequences cross-distributed. Isolates which have the totally same sequence, obtained from different cities or years, formed a cluster. However outbreak isolates which obtained from the same year 2015, are on different branches. These above suggest the complexity of measles transmission in Shandong: there are both single-chain transmission across year and multi-chain transmission in the same year in Shandong province. The comparative of outbreak and sporadic strains in evolution rate was made by the Bayesian Markov chain Monte Carlo (MCMC) method in BEAST, and the result showed the mean evolution rate of outbreak isolates and sporadic isolates was 1.585 N 10^− 3^ and 1.266 N 10^− 3^ substitutions per site per year, which is similar to the 1.19 N 10^− 3^ - 2.11 N 10^− 3^ evaluated by Xu et al. [19]. Hence, no clear difference was observed between outbreak and sporadic strains in evolution rate.

Viruses in the same transmission chain have identical or nearly identical N-450 sequences [10]. According to this criterion, together with the geographic dispersal result and Phylogenetic trees, we defined three main transmission chains in our study. The phylogeographic analysis displayed that outbreak cases and sporadic cases emerged alternatively and were reciprocal causationon on one transmission chain. Taking chain III for example, the case originated from Weihai (outbreak case) arrived Zaozhuang and induce the outbreak, then dispersed to Liaocheng (sporadic case) which seeded the outbreak case in Dezhou in 2016. Another interesting transmission chain (chain II), revealed that sporadic strains which could persisted for a certain long time may induce outbreak. On the chain II, sporadic cases in Jinan were reported in 2013. Years later, two outbreaks occurred in Weifang and Weihai in 2015 induced by virus strains which had the same amino acid sequence (only one difference in nucleotide sequences) with the sporadic cases in Jinan in 2013 on the chainII. So there may be certain significance to monitor genetic characteristics of sporadic cases as now we do to the outbreak cases in the worldwide.

As one important factor of measles transmission, vaccination history was also compared between the outbreak cases and sporadic cases in our study. All the 8 sporadic cases whose vaccination information was clear were inoculated with less than 2 doses, while for 8 outbreak cases whose vaccination information was available, only 1 case was inoculated with two doses. Due to limited number of patients in this study, credible statistical analysis was unable to be performed to investigate whether 2-dose inoculation can block the sporadic incidence of measles. However, the results in our study indicate that one or even 2-dose inoculation cannot completely block the measles outbreak yet, as some study reported previously. They observed that annual measles outbreaks with high vaccination rate also continued to occur [20, 21]. Although these outbreaks typically involved importation and mostly affect [22, 23], there were surprisingly high numbers of vaccine failure among one- and two-dose recipients of measles vaccine who were infected (approximately 2–12% for children immunized at/around 1 year of age [24–27].

This study has described the phylogenetic characterization and molecular evolution of measles virus in eastern China under the circumstance of accelerated global measles control. Cocirculation of multiple transmission lineages of measles virus were observed, and genetic divergence and evolution rate of outbreak and sporadic strains were investigated. The results highlight the importance of genetic analysis of measles virus on early monitoring and tracing of outbreaks.

## Conclusion

Based on the results of this study, we conclude that the monitoring of sporadic cases should focus on two points: i) supervision of transmission path, especially the path of those sporadic case induced by importation. ii) supervision of transmission time-scale. Continuous monitoring of virus strain with the same or similar sequences,can benefit the exploration whether the long-term existence of the same or similar strains is a high-risk factor for outbreaks. However, all the opinions above may be confined to this study, more research work should be proceeded employing more cases with more epidemic and genetic information.

## Material and methods

### Sample collection and virus isolate

A total of 15 measles outbreak cases and 18 measles sporadic cases were enrolled from Shandong measles surveillance system from 2013 to 2019. All the enrolled cases were defined according to the diagnostic criteria of measles. Throat swabs samples were collected from individuals identified as measles after obtaining informed consent from patients or their parents.

Clinical specimens were inoculated on Vero/SLAM (signaling lymphocytic activation molecule; also known as CDw150) cells [28] and the cells were observed for cytopathic effect (CPE) for 7 d. Inoculated cells were blind-passaged up to three times before being discarded. Cell cultures were harvested when typical CPE was observed.

### Sequence amplification and sequencing

All isolates were sequenced and genotyped using the standard protocols. Briefly, viral RNA was extracted from 140 μl of cell culture medium according to the manufacturer’s instructions (Omega, USA). RNA was reconstituted in 60 μl of nuclease-free water and stored at − 80 °C until tested. Measles virus N gene was amplified using primers MeV-F (5′-GGGAGGCTTGAACTTTGG-3′) and MeV-R (5′-TCCGTGTCTGAGCCTTGT-3′). One-step RT-PCR was performed using PrimeScript RT Master Mix (Takara, Dalian, China). The following PCR program was used: 50 °C for 30 min, 98 °C for 10 s; 98 °C for 1 s, 55 °C for 5 s, 72 °C for 10 s for 30 cycles;and a final extension step at 72 °C for 1 min. PCR products were purified using the Omega Gel Extraction kit. Sequences of the PCR products were obtained using BigDye terminator chemistry version 3.0 according to the manufacturer’s protocol for both sense and antisense strands on an automated ABI PRISM 3100 Genetic Analyzer.

### Sequence analysis

Sequences were analyzed using Sequencher 4.1.4 and aligned using BioEdit 7.0.5.3. Phylogenetic analyses were performed using the MEGA 7.0 [29, 30] software based on N-450 coding region. Phylogenetic trees were constructed by comparison with the reference strains defined by the WHO previously using the Maximum Likelihood method. The reliability of the groupings was estimated using bootstrap resampling of 1000 replicates. GenBank accession numbers of reference sequences used in this study were: LC002659, AB824116, AB915260, KF533141, KT031805, MH375617, MN602401, MK628225, and AB915260.

### Phylogenetic analysis using the Bayesian Markov chain Monte Carlo method

The evolution rate and molecular clock phylogeny of 33 Shandong isolates were inferred using the Bayesian Markov chain Monte Carlo (MCMC) method in BEAST version 1.6.1 [31]. The best nucleotide substitution model was selected using mega 7.0 [29, 30]. The clock model and demographic model were compared by calculating Akaike’s information criterion through MCMC (AICM) [32, 33], for each model using Tracer v1.6.1 [34]. In this study, we tested 4 clock models including a strict clock, an uncorrelated lognormal relaxed clock, an uncorrelated exponential relaxed clock, and a random local clock [35]. Two demographic models, constant size model and exponential growth model, were also compared using AICM calculation. We employed the model with the lowest AICM value. As a result, the datasets were analyzed using a GTR + G model under a strict clock with an constant size model. The MCMC chains were run for 30,000,000 steps and sampled every 10,000 steps. Convergence was assessed from the effective sample size (ESS) after a 10% burn-in using Tracer v1.6.1. ESS values above 200 were accepted. Uncertainty in the estimates was indicated by the 95% HPD intervals. The maximum clade credibility tree was generated by Tree Annotator v 1.6.1 after a 10% burn-in. The phylogenetic tree was viewed in FigTree v1.3.1. The evolutionary rates of outbreak sequences and sporadic sequences were also estimated as described.

### Phylogeographic analysis

The Bayesian Stochastic Search Variable Selection (BSSVS) was used to provide evidence for statistically supported diffusion between state variables under BEAST v1.6.1. The results of BSSVS was summarized using SPREAD v1.0.6 [36], a keyhole markup language (KML) file was generated to identify the major routes of geographic diffusion. Bayes factor (BF) test was used to select the most probable diffusion process. To visualize the geographic dispersal of the measles cases, the KML file was imported to Google™ Earth to produce a graphical animation of the estimated spatiotemporal pathways.

### Nucleotide sequence accession numbers

Newly generated N-450 sequences of Shandong measles virus isolates have been submitted to GenBank under the accession numbers OL807629-OL807661. All sequences data that support the findings of this study have been deposited in GenBank.

## Supplementary Information

**Additional file 1.** The XML file generated for the phylogenetic analysis with BEAST software.

**Additional file 2.** A graphical animation of the estimated spatiotemporal pathways of measles viruses produced by Google™ Earth.

**Additional file 3.** The KML file generated for the spatiotemporal analysis of measles viruses in this study.

## Data Availability

Newly generated N-450 sequences of Shandong measles virus isolates have been submitted to GenBank under the accession numbers OL807629-OL807661. All sequences data that support the findings of this study have been deposited in GenBank.

## Abbreviations

MeV: Measles virus
N-450: carboxy-terminal 450 nucleotides of N-region
SLAM: signaling lymphocyte-activation molecule
CPE: cytopathic effect
MEGA: Molecular Evolutionary Genetics Analyses
MCMC: Markov chain Monte Carlo
MRCA: The most recent common ancestor
HPD: highest posterior density
HKY: Hasegawa-KishinoYano
UCLD: uncorrelated lognormal-distributed
EG: exponential growth
