# Supplementary figures and images for: Comparative study on molecular epidemiology of measles H1 outbreak and sporadic cases in Shandong Province, 2013–2019
Source: BMC Genomics. 2022 Apr 14;23:305. doi: 10.1186/s12864-022-08492-x (PMC9011973; doi:10.1186/s12864-022-08492-x)

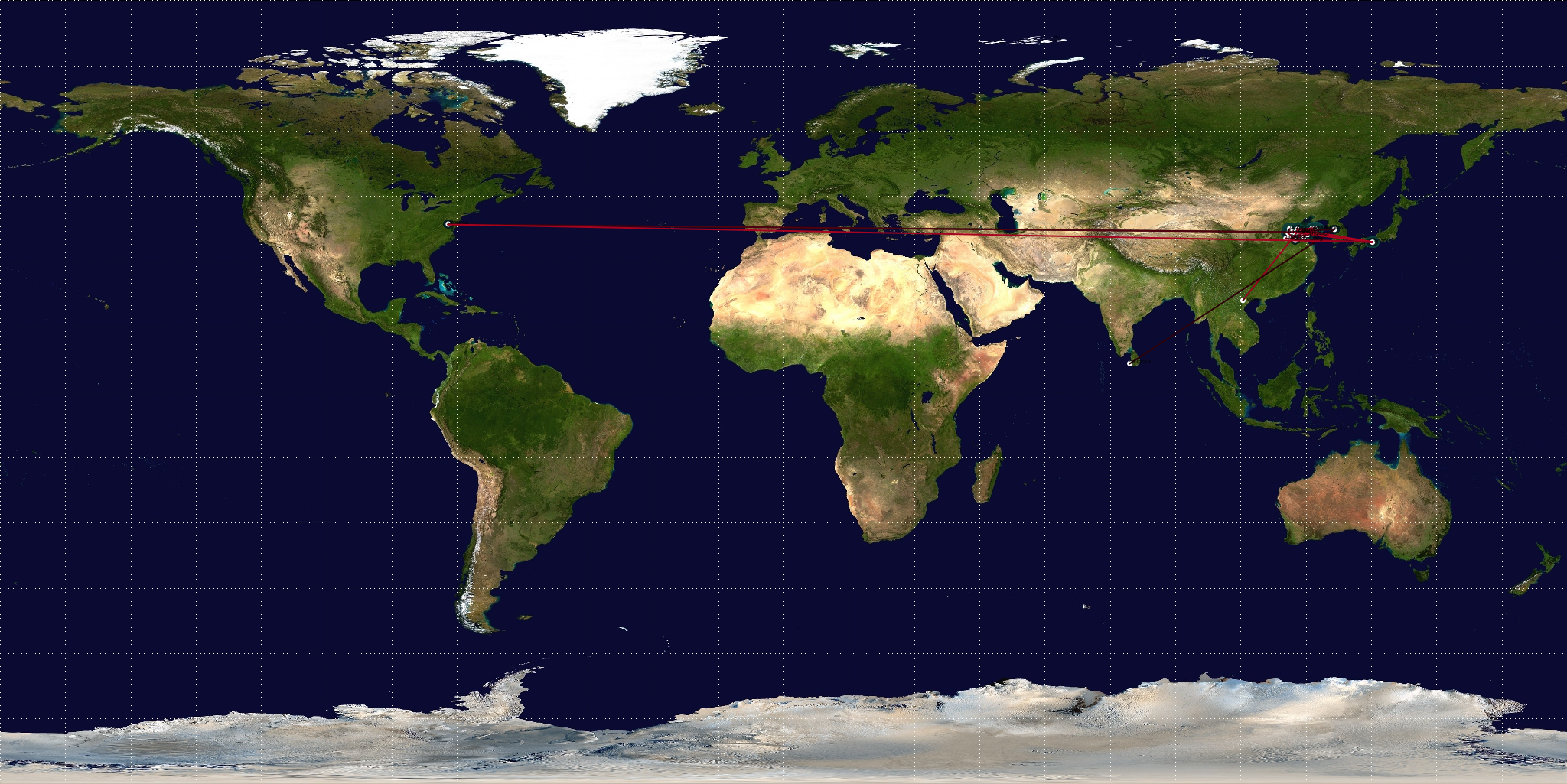

Supplement: Supplementary file 2 — Additional file 2. A graphical animation of the estimated spatiotemporal pathways of measles viruses produced by Google™ Earth. [file 12864_2022_8492_MOESM2_ESM.tif]
